# The Architecture of the Adhesive Apparatus of Cultured Osteoclasts: From Podosome Formation to Sealing Zone Assembly

**DOI:** 10.1371/journal.pone.0000179

**Published:** 2007-01-31

**Authors:** Chen Luxenburg, Dafna Geblinger, Eugenia Klein, Karen Anderson, Dorit Hanein, Benny Geiger, Lia Addadi

**Affiliations:** 1 Department of Molecular Cell Biology, Weizmann Institute of Science, Rehovot, Israel; 2 Department of Structural Biology, Weizmann Institute of Science, Rehovot, Israel; 3 Chemical Research Support Unit, Weizmann Institute of Science, Rehovot, Israel; 4 Infectious Diseases and Cell Adhesion Programs, The Burnham Institute for Medical Research, La Jolla, California, United States of America; Dresden University of Technology, Germany

## Abstract

**Background:**

Osteoclasts are bone-degrading cells, which play a central role in physiological bone remodeling. Unbalanced osteoclast activity is largely responsible for pathological conditions such as osteoporosis. Osteoclasts develop specialized adhesion structures, the so-called podosomes, which subsequently undergo dramatic reorganization into sealing zones. These ring-like adhesion structures, which delimit the resorption site, effectively seal the cell to the substrate forming a diffusion barrier. The structural integrity of the sealing zone is essential for the cell ability to degrade bone, yet its structural organization is poorly understood.

**Principal Findings:**

Combining high-resolution scanning electron microscopy with fluorescence microscopy performed on the same sample, we mapped the molecular architecture of the osteoclast resorptive apparatus from individual podosomes to the sealing zone, at an unprecedented resolution. Podosomes are composed of an actin-bundle core, flanked by a ring containing adhesion proteins connected to the core via dome-like radial actin fibers. The sealing zone, hallmark of bone-resorbing osteoclasts, consists of a dense array of podosomes communicating through a network of actin filaments, parallel to the substrate and anchored to the adhesive plaque domain via radial actin fibers.

**Significance:**

The sealing zone of osteoclasts cultured on bone is made of structural units clearly related to individual podosomes. It differs from individual or clustered podosomes in the higher density and degree of inter-connectivity of its building blocks, thus forming a unique continuous functional structure connecting the cell to its extracellular milieu. Through this continuous structure, signals reporting on the substrate condition may be transmitted to the whole cell, modulating the cell response under physiological and pathological conditions.

## Introduction

Bone is a mineralized tissue with an ordered composite structure consisting of arrays of collagen fibers with inter-grown carbonated hydroxyapatite crystals. With maturation, the relative proportion of mineral increases and modifies the material properties of bone [1]. To preserve the tissue integrity and in response to physiological stimuli, bone is continually remodeled through the concerted activity of bone resorbing cells, the osteoclasts, which dissolve mature bone, and bone depositing cells, the osteoblasts, which lay down new bone. The complementary roles of these two cell types are thus central to the development, ordered growth and damage repair of the skeleton [2].

Osteoclasts are large multinucleated, highly motile and invasive cells of hematopoietic origin, which alternate between migratory and bone-resorbing stages. Osteoclast mediated bone resorption is tightly regulated at multiple levels [3], [4]. Excessive osteoclast activity results in progressive loss of bone mass and deterioration of bone architecture, leading to a variety of diseases, the best known of which is osteoporosis, a major public health problem affecting growing numbers of postmenopausal women and older men [5].

The resorptive activity of osteoclasts depends on their adhesion to the bone surface. The primary adhesion mediating structures of osteoclasts are dot-like actin-rich structures known as podosomes [6]. Podosomes are highly dynamic structures formed not only in osteoclasts, but also in other monocyte-derived cells, such as macrophages and dendritic cells, as well as in smooth muscle cells, endothelial cells, transformed fibroblasts and certain epithelial cells [7]. Based on fluorescence and EM studies [7]–[11], podosomes were shown to consist of a central core of actin filaments, ∼0.3 µm in diameter and 0.6–1 µm high, surrounded by a ‘cloud’ of F-actin [12] and actin monomers [13], along with a variety of actin-associated proteins that regulate actin nucleation, severing, bundling and dynamics [14], [15]. Dynamin is associated with a thin membrane invagination in the actin core center [16]. Around the core bundle, there is a peripheral ring with typical diameter of ∼1 µm, containing integrins and a variety of associated adaptor and signaling proteins including paxillin, vinculin, talin and different kinases [17]–[20]. The architecture of the central core and the plaque, and in particular their connections one with the other, have not been elucidated at high resolution.

In cultured osteoclasts, podosomes undergo major reorganization during their maturation. From individual structures, scattered throughout the cell ventral membrane, via ordered clusters, they develop rings, which extend towards the cell periphery, merge with neighboring rings and eventually stabilize, forming a belt of podosomes or further condensing into a sealing zone-like structure [21]. Similar patterns were observed in cells plated on degradable substrate [11], [15], [22], [23]. Abnormal podosomes were shown in several animal models and in humans, to inhibit bone-resorption [24].

In bone-associated osteoclasts, the sealing zone is essential for the degradation process, as protons and lysosomal enzymes are secreted into the sealed space between the cell and the bone surface, degrading the mineral and collagen components, respectively [25], [26]. Recently, Chiusaroli et al [27] showed that when defective sealing zones are formed, the osteoclast ability to degrade bone in vitro and in vivo is impaired. The accurate structural organization of the sealing zone is thus essential for bone resorption. The spatial-temporal relation between the different podosome-based structures in cultured and bone attached osteoclasts is still unclear.

In order to obtain a comprehensive understanding of the osteoclast adhesive apparatus, visualization of large cellular structures, such as podosomes, must be performed simultaneously with the visualization of fine molecular details, such as individual actin filaments. Immunolabeling of multiple components or live cell dynamic recording can be readily accomplished using fluorescence microscopy, while retrieval of high resolution structural data requires advanced electron microscopy. In this study we used correlated fluorescence and high resolution scanning electron microscopy performed on the same osteoclasts cultured on artificial substrates and on bone. The result is a description at an unprecedented resolution of podosome assembly, from individual units to clusters and sealing zone peripheral belt.

## Results

Osteoclast precursor RAW264.7 cells were cultured on glass slides or on electron microscope grids, and induced to differentiate. Mature osteoclasts were plated directly on bone. RAW-derived osteoclasts are bone degrading cells conforming to all the conventional assays for osteoclast characterization, and having the same cytoskeleton/adhesion organization as primary osteoclasts [12], [20], [21], [28].

The osteoclast cell body was mechanically removed, leaving only the cell ventral membrane attached to the substrate, with its adhesive apparatus and the associated cytoskeleton intact. The cell preparations were then labeled and inspected, sequentially, by fluorescence microscopy and high resolution scanning electron microscopy.

**Figure 1:**
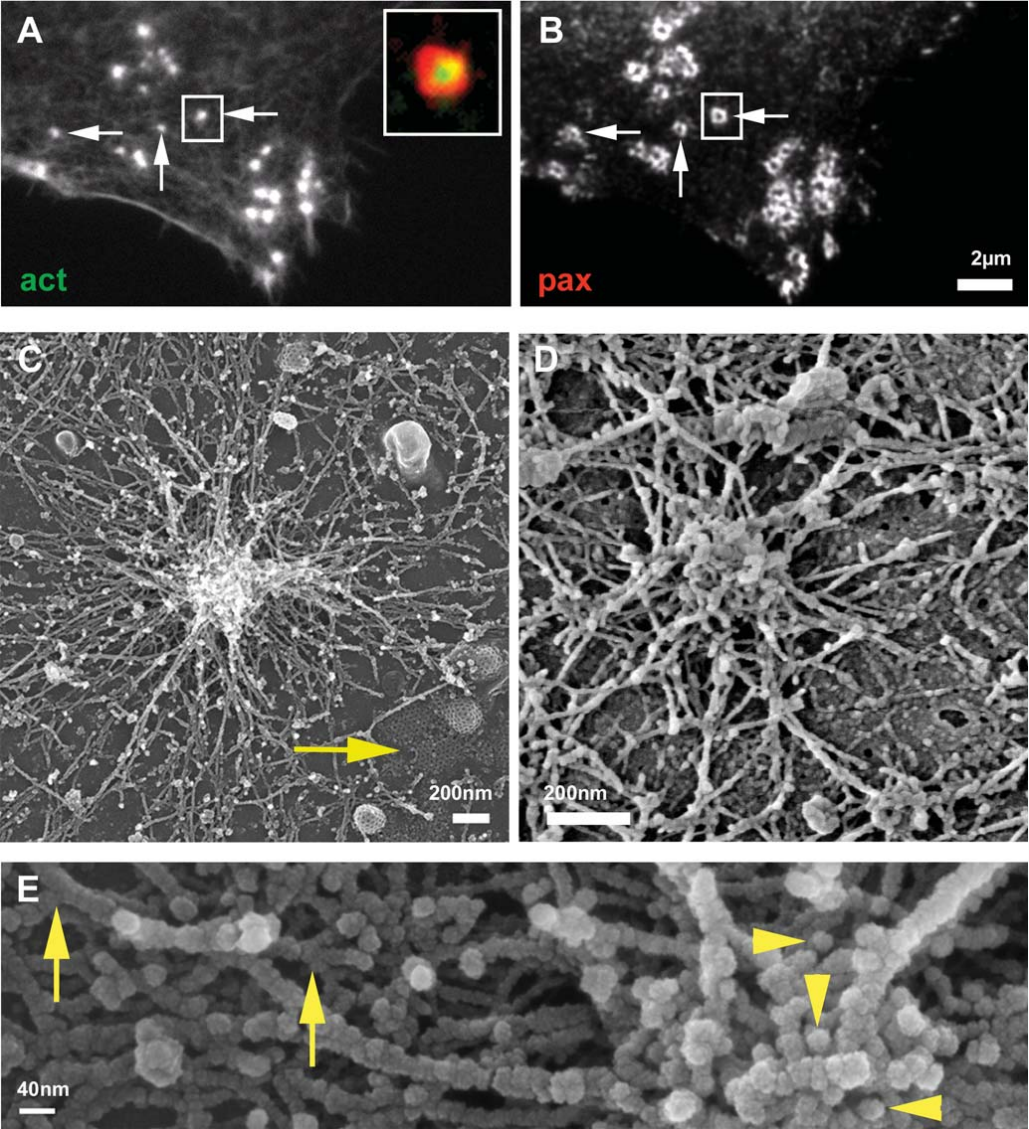
Individual podosome. Intact osteoclast (A,B) or ventral membranes (C–E) fixed and stained for actin (A) or paxillin (B) or prepared for HR-SEM (C, D, E). Individual podosomes (A, B arrows) are isolated from nearby structures and show no overlap with neighboring podosomes in paxillin staining (B, arrow). The insert in (A) shows a merged image of actin/paxillin staining. (C) An isolated podosome showing a dense actin core at the center of the podosome and actin fibers radiating from it and interacting with the ventral membrane. Note membrane-associated clathrin pits (C, arrow) testimony of the excellent preservation of the sample. (D) Structures very similar to (C) are detected in osteoclasts plated on bone slices. (E) Higher magnification of a podosome shows tightly packed actin fibers at the actin core, many of which are almost perpendicular to the substrate (arrowhead). The occasionally observed concavity at the peak of the podosome (D,E) may reflect the underlying membrane invagination related to dynamin (16). The actin fibers radiating from the core show branching points (arrows).

Individual podosomes are seen in fluorescence microscopy as isolated adhesion units [21] (Fig 1A,B arrow), with distinct actin cores and ring domains that are separated from those of other podosomes. When observed by scanning electron microscopy (SEM, Fig 1C,D), isolated podosomes consist of a densely packed, ∼300 nm thick core bundle of distinct actin fibers, pointing in a direction more or less perpendicular to the membrane (Fig 1E, arrowheads). A dome-like radial network of less densely packed fibers extends from the core and appears to anchor the core to the membrane. The circumference of the isolated podosomes, up to ∼3 µm in diameter, is clearly defined as an area where the actin fibers merge with the membrane. This anchor zone is further surrounded by randomly oriented cortical-membranal actin fibers that are found throughout the entire cytoplasmic face of the ventral membrane (Fig 1C, D).

A similar structural organization of individual podosomes was observed in osteoclasts on all substrates used in this study: glass, EM grids and bone (Fig 1D), as well as in macrophages (Supp. Fig S1) [29]. These results indicate that a basic structure is characteristic of podosomes in general, independent of substrate or cell type.

**Figure 2:**
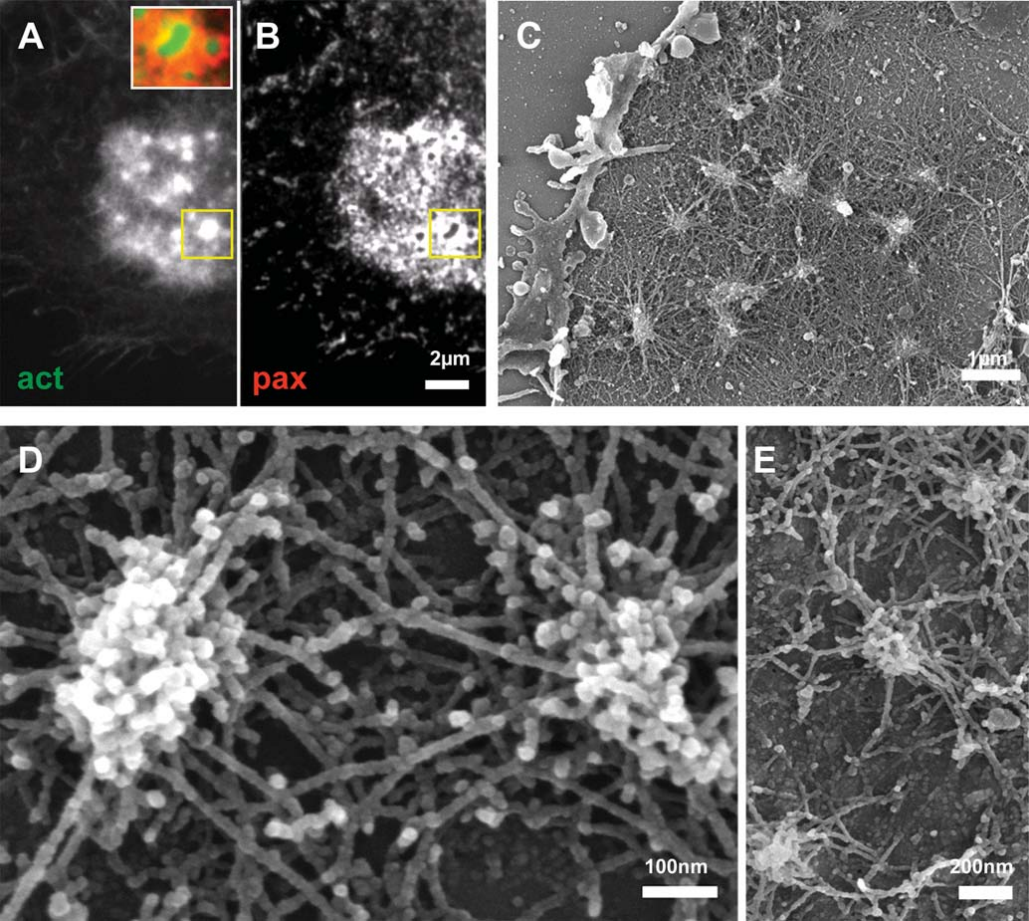
Clustered podosomes. Intact osteoclast (A,B) or ventral membranes (C–E) fixed and stained for actin (A) or paxillin (B) or prepared for HR-SEM (C–E). (A) Actin pillars emanate from an “actin-cloud”. (B) Paxillin is largely shared by the cluster and overlaps with the “actin-cloud”. The insert in (A) shows a merged image of actin/paxillin staining. (C) SEM over-view of a cluster. The radial podosome actin fibers appear to correspond to the “actin-cloud”. (D) Higher magnification view of two clustered podosomes showing interactions between radial peripheral cables. There are no cables directly connecting between cores. (E) The same organization as in (D) is detected on bone.

When individual podosomes develop into clusters by means of fission [21], [29], the so-called actin cloud (Fig 2A), surrounding the podosome cores (Fig 2A) becomes prominent. Quantitative fluorescence microscopy analysis shows that the amount of actin associated with the podosome core is typically ∼2–3 folds higher then that associated with the actin “cloud”. The ring domains of neighboring podosomes apparently fuse or overlap, and co-localize with the actin cloud (Fig 2B). The basic podosome architecture remains the same as that observed in isolated podosomes (Fig 2C, D). In contrast to the individual podosome, however, the radial fibers of adjacent podosomes meet at the podosome periphery (Fig 2 D), creating a dense actin network surrounding the cluster (Fig 2C). There are, however, no fibers directly connecting neighboring cores. Correlated high resolution SEM/fluorescence microscopy of a cluster confirms that the radial networks between neighboring cores, correspond to the ‘actin cloud’ observed by fluorescence (Supp. Fig S2).

Morphometric analysis of podosome spacing in osteoclasts plated on glass, yields a typical distance (core-to-core) between clustered podosomes of 750±170 nm (n = 112 podosome pairs from 4 different cells). The overall morphology of podosome clusters in osteoclasts plated on bone or EM grids is similar to that observed on bone (Fig 2E).

**Figure 3:**
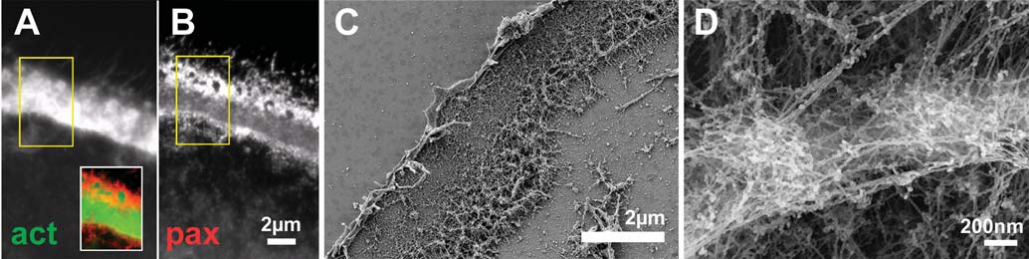
Sealing zone-like structure. Intact osteoclast (A,B) or ventral membrane (C, D) on glass cover-slips were fixed and stained for actin (A) or paxillin (B) or prepared for HR-SEM (C–D). (A) A dense actin belt is surrounded by inner and outer paxillin belts (B). The insert in (A) shows a merged image of actin/paxillin staining. (C) SEM over-view of a sealing zone-like structure on glass: note the continuous, robust actin organization. (D) Higher magnification view of two neighboring pillars showing numerous inter-pillar cables.

**Figure 4:**
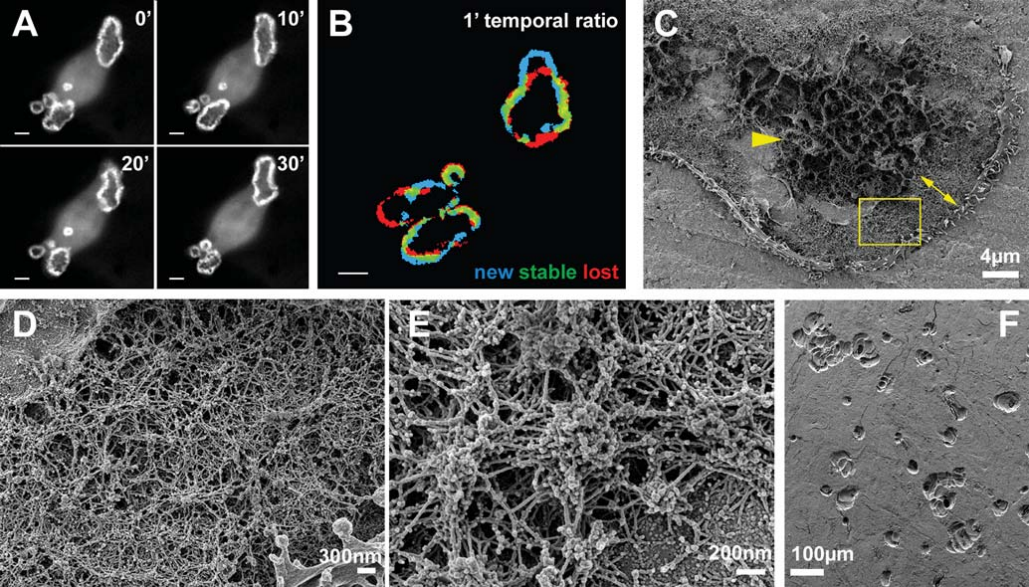
Sealing zone. (A, B) Frames from a movie taken by time-lapse microscopy from live GFP-actin osteoclasts plated on bone. (A) Four frames taken at 10 minutes intervals showing dynamic reorganization of actin belts. (B)One minute temporal ratio figure. Blue pixels represent new structures and red pixels faded structures. Note the high rate of dynamic reorganization. (C–E)Osteoclast ventral membrane on bone. (C) SEM overview of the ventral membrane of a cell plated on bone. The central area (arrow head) presumably corresponds to the ruffled border. The sealing zone (double arrow) is thicker than on glass. (D, E) Higher magnification views (from yellow box in (E)) of the podosomes forming the sealing zone. (F) Osteoclasts were plated on bone slices and removed three days later, leaving numerous resorption pits on the surface.

A drastic reorganization in architecture is observed when podosomes develop into the sealing zone-like or sealing zone structures (Fig 3 and 4, respectively). When plated on artificial substrates such as glass or EM grids, the sealing zone-like structure appears as a ∼2–3 µm wide actin belt surrounded by inner and outer plaque domains (Fig 3A–C). Quantitative fluorescence microscopy analysis shows that the actin intensity associated with the cores is now higher by only 20–40% compared with the actin intensity associated with the “cloud” located between neighboring podosomes. Podosome cores are, however, still detectable with the same basic architecture of an actin core and radial fibers (Fig 3 C, D). However, these podosomes are highly interconnected. Multiple actin fibers connect adjacent podosome cores at all heights (Fig 3D), while the average distance between podosome cores is reduced to 480±140 nm (n = 197 podosome pairs from 6 cells). Densely distributed actin fibers radiate from the podosome core, establishing contact with the membrane (Fig 3C).

Interestingly, the density of fibers docking the structure to the ventral membrane is much higher on the side of the ring facing the outer plaque, relative to the inner side. This correlates well with the plaque fluorescence being much more intense in the external plaque domain (Fig 3A, B). The plaque is clearly shared between podosomes.

Bona fide sealing zones of polarized osteoclasts plated on bone (Fig 4A–F) are often associated with underlying resorption pits (Fig 4F). The ventral membranes of these cells are characterized by a highly convoluted membrane around the cell center, distinctly different from that found in cells growing on glass or EM grids (Fig 4C arrowhead). This area presumably corresponds to the ruffled border, which is involved in the secretion of acid and proteolytic enzymes at the cell-bone interface [2], [30].

The actin belt in the sealing zone of bone resorbing osteoclasts is 3–6 µm wide, much thicker than that formed by cells cultured on glass (Fig 4C double headed arrow) and the actin network is considerably denser (Fig 4D, E). However, the basic architecture is clearly preserved. It consists of podosomal units, including F-actin pillars perpendicular to the substrate, connected by numerous inter-pillar filaments, running parallel to the substrate. The average distance between actin cores in these cells is further reduced to 210±50 nm (n = 400 podosome pairs from 5 cells). Sealing zones of bone resorbing osteoclasts are highly dynamic (Fig 4A, B, Movie S1) suggesting high levels of actin remodeling [21].

**Figure 5:**
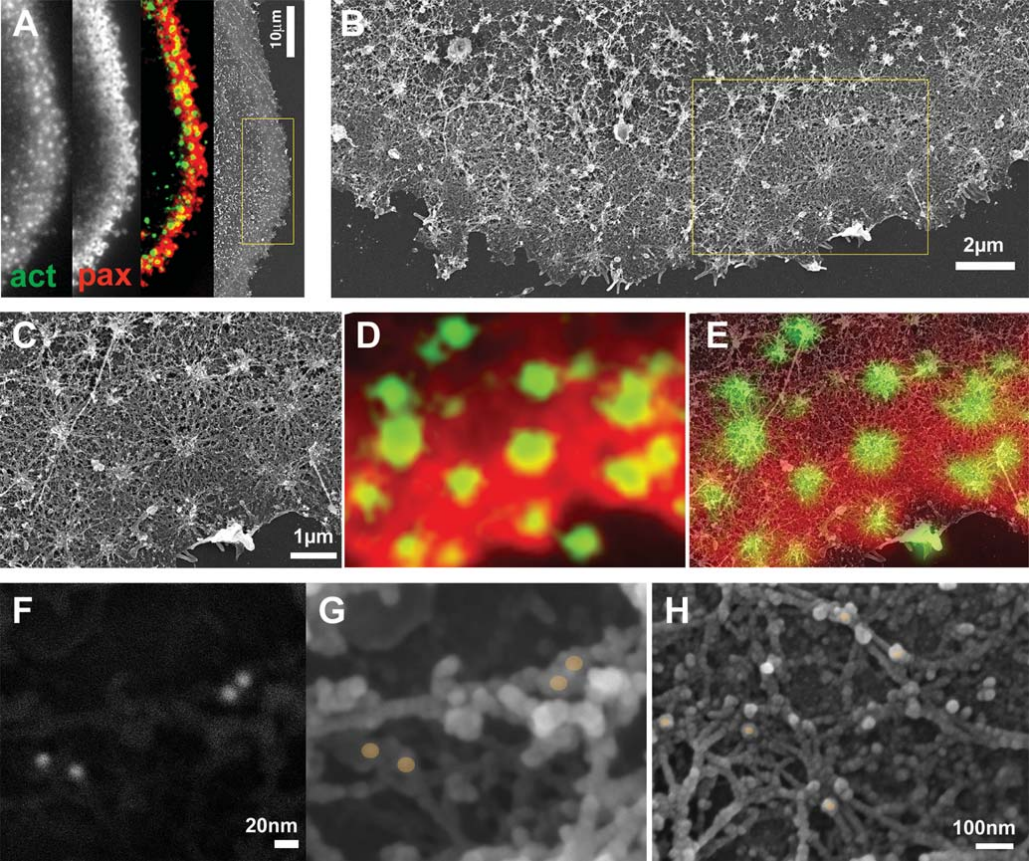
Structural relations between podosome ring and core domains. (A) Osteoclast ventral membranes were labeled for paxillin and actin, and simultaneously prepared for HR-SEM. On the left is the actin labeling, followed by paxillin labeling and by their merged picture. On the right is the corresponding SEM micrograph. (B) Higher magnification view of the area in the yellow rectangle in (A). (C) Higher magnification view of the area in the yellow rectangle in (B). (D) Merged actin/paxillin labeling from the same area as in (C). (E) Merged image between (C) and (D). The correlation between SEM and immunofluorescence shows paxillin association with podosome radial actin fibers, reaching up to but not co-localizing with the central bundle. (F, G, H) Osteoclast ventral membranes were labeled for paxillin with 15nm colloidal gold particles and visualized by back scattering signal (F) or secondary electron detector (G, H). (F) and (G) show the same area. The colloidal gold particles in (G, H) are marked with brownish dots, showing paxillin in association with actin fibers in close proximity with the ventral membrane.

To obtain a quantitative molecular insight into the organization of podosomes and podosome super-structures we have correlated fluorescence labeling patterns of ventral membranes by using light microscopy and high resolution SEM performed on the same samples. Ventral membranes, double-labeled for actin and paxillin were first examined by light microscopy, then critical-point dried and examined by SEM. Superposition of the fluorescence and SEM images shows that intense actin fluorescence is associated with the actin cores visualized by SEM, while the “actin cloud” corresponds to the radial actin cables, inter-connecting neighboring podosomes (compare Fig 5A to B, C); the plaque domains extend under the actin cloud, reaching up to the core (Fig 5C,D,E). The extraordinary state of preservation of the membrane allowed mapping of the location of the plaque protein paxillin also by immuno-gold labeling. The location of the protein was indicated by the strong back scattering signal of the colloidal gold particles (Fig 5F), and was then localized in the secondary electron images, relative to the other components (Fig 5G, H). In all states of podosome organization paxillin immuno-gold labeling was closely associated with actin fibers, either at fiber crossings or directly in contact with the cell membrane. The exclusive co-localization of paxillin and actin at sites in close proximity with the ventral membrane suggests a direct interaction between podosome-associated actin and the plaque. This observation has far reaching implications to osteoclast activity, in particular to osteoclast communication and sensing of the substrate extracellular milieu, because it suggests that any modification in intra- or extra-cellular architecture is directly transmitted and sensed through the communication network by the whole structure.

## Discussion

The results reported here provide insight into the mechanism underlying the assembly of the sealing zone, the adhesion apparatus and diffusion barrier of bone resorbing osteoclasts. The spatial and temporal relations between podosomes and the sealing zone are controversial. While it has been suggested that isolated podosomes fuse to give rise to a continuous sealing zone [23], [31]–[33] there have also been claims that the sealing zone on bone has a different three-dimensional organization that is not derived from podosomes [28], [34]. Here, we show that the sealing zone on bone is made of structural units clearly related to individual podosomes, which differ mainly in their densities and degree of inter-connectivity from the podosome belt observed on glass. It appears that while the elementary adhesion units are preserved all along the process of osteoclast maturation, there is a progressive process of consolidation of the podosomal scaffold, provided by the radial actin filaments, which initially anchor the core bundle to the neighboring membrane, and later also interconnect between podosome units. Whether this process is responsible for the progressive condensation of podosome cores, or is a consequence thereof is not clear yet.

The results presented here, together with information derived from our previous studies [20], [21], are suggestive of a mechanism where podosomes proliferate and transform from individual dynamic structures, that can nucleate sporadic adhesions, to 2-dimensional arrays (“clusters”); from these much more highly dynamic rings are formed, which eventually stabilize. This transition correlates with enhanced actin reorganization and a 10-fold increase in the amounts of actin associated with the podosome unit, as well as a similar increase in the plaque protein levels [20], [21]. As the podosome units remain largely unchanged, these increased rates and increased local actin levels are most likely attributable to the formation of an interconnecting actin network and to an increase in actin-core height. Interestingly, the intensity of the actin cloud relative to the core increases by a factor of 2–3 between the cluster and the sealing zone-like podosomes, indicating that the relative increase in the local levels of actin associated with the radial, inter-pillar fibers is even more dramatic (in the order of >20). This increase in actin cloud intensity, in addition to the shorter inter-pillar distance in the sealing zone, makes it virtually impossible to resolve individual podosome units by light microscopy.

We conclude that the podosome scaffold is the fundamental structural unit that provides the infrastructure directing the new wave of actin polymerization. Podosomes are thus preserved as fundamental building blocks at all stages of osteoclast development, adhesion and resorption activity, highlighting their key role in osteoclast function and malfunction.

The intimate structural relations between the plaque domain and the actin structures in individual podosomes and especially in the tightly interconnected sealing zone suggest that the local properties of the substrate may be transmitted through the adhesion receptors to the adhesion plaque proteins and then to the continuous actin network. The whole structure, including the adhesion plaque and the actin ring, would then operate as one functional unit, which can potentially sense the condition of the substrate and react to it accordingly, thus effectively regulating the resorption activity.

## Materials and Methods

### Tissue culture, substrates and antibodies

RAW 264.7 cells were from the American Type Culture Collection (ATCC) (Manassas, VA, USA). To induce osteoclast differentiation 100 cells/mm^2^ were plated on EM nickel grids (SPI, West Chester, PA, USA) or glass cover-slips in alpha MEM with Earle's salts, L-glutamine and NaHCO_3_ (Sigma Chemical Co., St. Louis, MO, USA) supplemented with 10% fetal bovine serum (FBS) (Gibco, Grand Island, NY, USA) antibiotics (Biological Industries, Beit Haemek, Israel) 20 ng/ml recombinant soluble Receptor activator of NF kappa B ligand (RANK-L) and 20 ng/ml Macrophage colony-stimulating factor (mCSF) (R&D, Minneapolis, MN, USA), at 37°C in a 5% CO_2_ humidified atmosphere for 3 days. For experiments on bone, RAW cells were induced to differentiate in plastic dishes as described, removed with 10 mM EDTA for 15min. and plated for 30 hours on thin slices mechanically sawed from cattle femurs and kept in PBS prior to plating.

IC21 macrophages were from ATCC. These cells were cultured in RPMI 1540 (Gibco, Grand Island, NY, USA) supplemented with 10% FBS and 10ng/ml M-CSF.

Primary antibody: monoclonal anti paxillin (BD., San Jose, CA, USA). Secondary antibody: goat anti mouse IgG, conjugated to cy3 (Jackson ImmunoResearch Laboratories Inc., West Grove, PA, USA). Actin was labeled with phalloidin, conjugated to FITC (Sigma Chemical Co., St. Louis, MO, USA).

### Fluorescence microscopy

Cells were fixed for 2 min in warm 3% paraformaldehyde (PFA) (Merck, Darmstadt, Germany)+0.5% Triton X-100 (Sigma Chemical Co., St. Louis, MO, USA) and then in PFA alone for additional 40 minutes. After fixation cells were washed 3 times with PBS, pH7.4 and incubated with primary antibody for 40 min, washed again 3 times in PBS and incubated for additional 40 min with the secondary antibodies.

For live cell imaging RAW cells stably expressing GFP-actin [21] were induced to differentiate in plastic dish and then plated on bone slices, as described.

All image analyses were carried out using Priism software for Linux operating systems (http://msg.ucsf.edu/IVE/Download/).

Temporal ratios were produced as previously described [35]. Briefly, the temporal ratio frame t+x was divided by t to produce a “spectral image” in which blue pixels indicated new positive pixels, red pixels indicated faded structures and the intermediate colors represented more stable structures.

Data were acquired with a DeltaVision system (Applied Precision Inc., Issaquah, WA, USA), consisting of an inverted microscope IX70 equipped with 60x/1.4 or 100x/1.3 objective (Olympus, Tokyo, Japan). The grey-scale wide-field pictures shown here are without any image filtration or manipulation.

### Ventral membrane preparation (VMP) for SEM microscopy

We developed a sample preparation technique for high-resolution three-dimensional correlative fluorescence and electron microscopy characterization of adhesive structures in cellular environment, which we coined VMP for ventral membrane preparations (Anderson, Page, Beck, Nickell, Volkmann and Hanein, manuscript in preparation). The procedure is an adaptation of published procedures [10], [36], which involves unroofing the cell's basal portion while the components preserve their three-dimensional organization and immunogenicity.

For the SEM samples, following the VMP preparations, cells were immediately fixed with warm 2% gluteraldehide (GA) (EMS, Hatfield, PA, USA) in PBS for 30 min. Cells were then washed 3 times for 5 min. in PBS, and twice with cacodylate buffer (0.1MCaCO, 5mMCaCl pH7.3) (Merck, Darmstadt, Germany), post-fixed with 1% OsO_4_ (EMS, Hatfield, PA, USA) for 45 min, washed 3 times in cacodylate buffer and then twice with H_2_O; the preparations were then incubated with1% tannic acid (Merck, Darmstadt, Germany) for 5 min., washed 3 times in H_2_O, incubated with 1% Uranyl Acetate (EMS., Hatfield, PA, USA) for 30min, and washed 3 times with H_2_O. Dehydration in increasing concentrations of reagents grade ethanol (2×5 min.for 25%, 50%, 70%, 95% and 2×10 min. for 100%) was followed by critical point drying using CPD30 (BAL TEC., Blazers, Lichtenstein), coated with 1–2 nm Cr using K575X (Emi Tech., Kent, England). The samples were visualized in the high-resolution SEM Ultra 55 (Zeiss, Oberkochen, Germany). For bone slices, the procedure was the same as for glass, excluding the incubation with Uranyl acetate.

### Immuno-gold-labeling

For Immuno-gold-labeling VMPs were prepared as described and fixed with 3% PFA+0.05% GA in PBS. Samples were washed 3 times in PBS+0.1%Glycine (Sigma Chemical Co., St. Louis, MO, USA) and then incubated in blocking solution: 0.1%Glycine, 0.1%Gelatine, 0.1%BSA, 0.1%twin20 (all from EMS, Hatfield, PA, USA) for 5min. The preparations were then incubated with primary antibody overnight at 4°C, samples were washed as described and incubated with 15 nm colloidal gold secondary goat anti mouse antibody (EMS., Hatfield, PA, USA) for 1 hour. The samples were washed again 5 times in PBS, fixed in 0.05%GA for 15min., post fixed and prepared for the EM as described above.

## Supporting Information

Figure S1 Macrophages podosomes. Ventral membranes of IC21 macrophages were prepared for SEM. (A) An over-view of macrophage. (B) Higher magnification view of podosomes at the leading edge of the cell. Note the similarity in podosome organization to osteoclasts.
(1.99 MB TIF)

Figure S2 Relation between podosome radial fibers. Osteoclast ventral membranes were labeled for paxillin and actin, and simultaneously prepared for HR-SEM. (A) an over-view of a cluster of podosomes, visualized under the HR-SEM. (B) fluorescence labeling of actin (green) and paxillin (red). (C) Merged image between (A) and (B). Note paxillin colocalization with podosome radial actin fibers.
(2.63 MB TIF)

Movie S1 Sealing zone dynamics of a resorbing osteoclast. Movie taken on live GFP-actin osteoclasts plated on bone. Frames were recorded at 1 minute intervals by time-lapse microscopy for 3.5 hours.
(0.98 MB MOV)
