# Supplementary figures and images for: The Architecture of the Adhesive Apparatus of Cultured Osteoclasts: From Podosome Formation to Sealing Zone Assembly
Source: PLoS One. 2007 Jan 31;2(1):e179. doi: 10.1371/journal.pone.0000179 (PMC1779809; doi:10.1371/journal.pone.0000179)

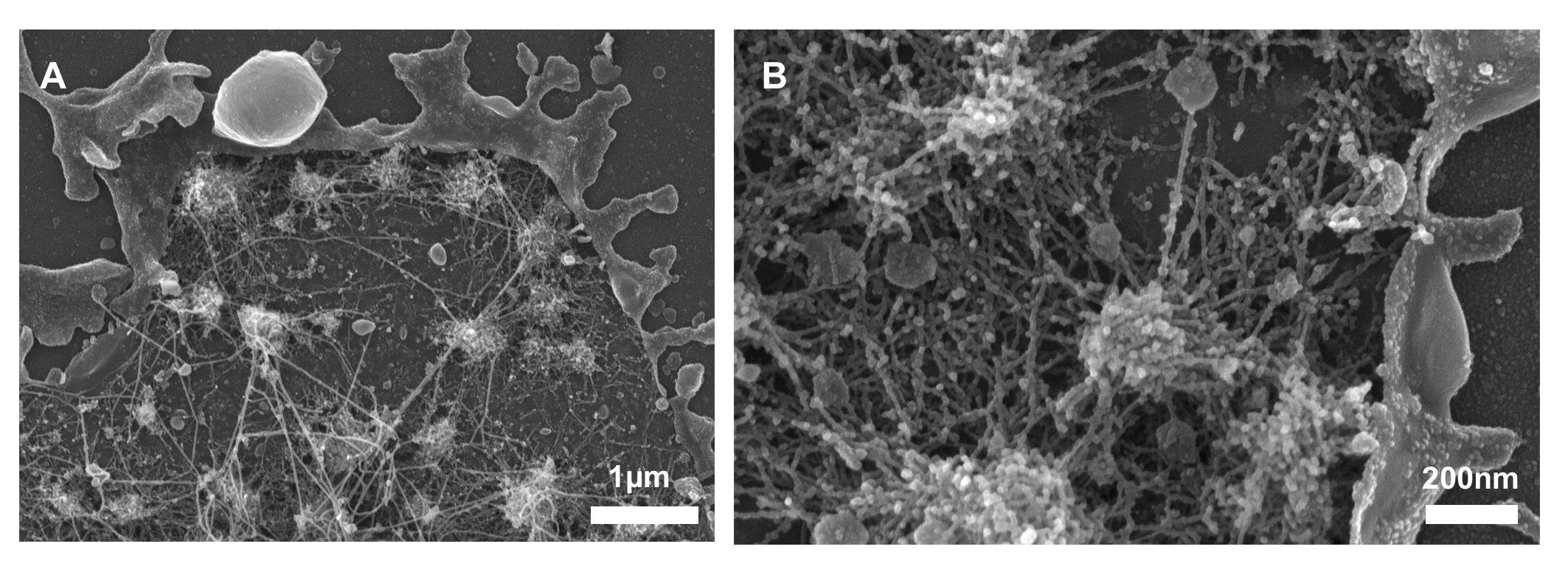

Supplement: Figure S1 — Macrophages podosomes. Ventral membranes of IC21 macrophages were prepared for SEM. (A) An over-view of macrophage. (B) Higher magnification view of podosomes at the leading edge of the cell. Note the similarity in podosome organization to osteoclasts. (1.99 MB TIF) [file pone.0000179.s001.tif]

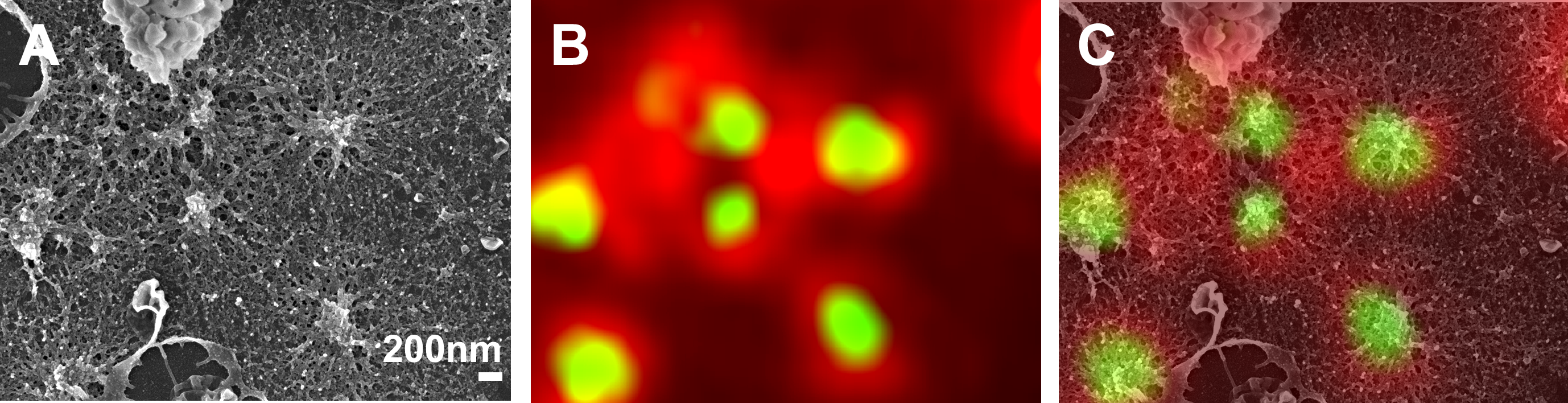

Supplement: Figure S2 — Relation between podosome radial fibers. Osteoclast ventral membranes were labeled for paxillin and actin, and simultaneously prepared for HR-SEM. (A) an over-view of a cluster of podosomes, visualized under the HR-SEM. (B) fluorescence labeling of actin (green) and paxillin (red). (C) Merged image between (A) and (B). Note paxillin colocalization with podosome radial actin fibers. (2.63 MB TIF) [file pone.0000179.s002.tif]
